# Supplementary material for: Lost to follow-up and associated factors among patients with drug resistant tuberculosis in Ethiopia: A systematic review and meta-analysis
Source: PLoS One. 2021 Mar 18;16(3):e0248687. doi: 10.1371/journal.pone.0248687 (PMC7971507; doi:10.1371/journal.pone.0248687)
Supplement: S1 File — (DOCX) [file pone.0248687.s001.docx]

# **Search strategies**

**PubMed:**

((((((((("treatment outcome"[MeSH Terms] OR ("treatment"[All Fields] AND "outcome"[All Fields])) OR "treatment outcome"[All Fields]) OR ("treatment"[All Fields] AND "outcomes"[All Fields])) OR "treatment outcomes"[All Fields]) OR ((("poverty"[MeSH Terms] OR "poverty"[All Fields]) OR "poor"[All Fields]) AND (("treatment outcome"[MeSH Terms] OR ("treatment"[All Fields] AND "outcome"[All Fields])) OR "treatment outcome"[All Fields]))) OR ((((((("therapeutics"[MeSH Terms] OR "therapeutics"[All Fields]) OR "treatments"[All Fields]) OR "therapy"[MeSH Subheading]) OR "therapy"[All Fields]) OR "treatment"[All Fields]) OR "treatment s"[All Fields]) AND ((((("default"[All Fields] OR "defaulted"[All Fields]) OR "defaulter"[All Fields]) OR "defaulters"[All Fields]) OR "defaulting"[All Fields]) OR "defaults"[All Fields]))) OR (((("lost to follow-up"[MeSH Terms] OR ("lost"[All Fields] AND "follow up"[All Fields])) OR "lost to follow up"[All Fields]) OR (("lost"[All Fields] AND "follow"[All Fields]) AND "up"[All Fields])) OR "lost to follow up"[All Fields])) OR (("unfavorable"[All Fields] OR "unfavourable"[All Fields]) AND (("treatment outcome"[MeSH Terms] OR ("treatment"[All Fields] AND "outcome"[All Fields])) OR "treatment outcome"[All Fields]))) AND (((((("tuberculosis, multidrug-resistant"[MeSH Terms] OR ("tuberculosis"[All Fields] AND "multidrug resistant"[All Fields])) OR "multidrug-resistant tuberculosis"[All Fields]) OR ((("multi"[All Fields] AND "drug"[All Fields]) AND "resistant"[All Fields]) AND "tuberculosis"[All Fields])) OR "multi drug resistant tuberculosis"[All Fields]) OR (((("tuberculosis, multidrug-resistant"[MeSH Terms] OR ("tuberculosis"[All Fields] AND "multidrug resistant"[All Fields])) OR "multidrug-resistant tuberculosis"[All Fields]) OR ("mdr"[All Fields] AND "tb"[All Fields])) OR "MDR-TB"[All Fields])) OR (((("tuberculosis, multidrug-resistant"[MeSH Terms] OR ("tuberculosis"[All Fields] AND "multidrug resistant"[All Fields])) OR "multidrug-resistant tuberculosis"[All Fields]) OR (("drug"[All Fields] AND "resistant"[All Fields]) AND "tuberculosis"[All Fields])) OR "drug resistant tuberculosis"[All Fields]))) AND (((((((((((("ethiopia"[MeSH Terms] OR "ethiopia"[All Fields]) OR "ethiopia s"[All Fields]) OR "Tigray"[All Fields]) OR "Afar"[All Fields]) OR "Amhara"[All Fields]) OR "Oromia"[All Fields]) OR ("somali"[All Fields] OR "somalis"[All Fields])) OR ("Benishangul"[All Fields] AND "Gumuz"[All Fields])) OR "Gambella"[All Fields]) OR (("southern"[All Fields] OR "southerns"[All Fields]) AND (("ethiopia"[MeSH Terms] OR "ethiopia"[All Fields]) OR "ethiopia s"[All Fields]))) OR ("Dire"[All Fields] AND "Dawa"[All Fields])) OR "Harar"[All Fields])

**Hinari:**

((lost to follow-up) OR (treatment outcome) OR (poor treatment outcome) OR (default)) AND ((multi-drug resistant tuberculosis) OR (drug resistant tuberculosis) OR (MDR-TB)) AND (Ethiopia) humans 2009-2020

**Google scholar:** (allintitle: ("treatment outcome" OR "lost to follow up" OR default) AND ("multi drug resistant tuberculosis" OR "MDR-TB") AND Ethiopia)
