# Supplementary material for: Lost to follow-up and associated factors among patients with drug resistant tuberculosis in Ethiopia: A systematic review and meta-analysis
Source: PLoS One. 2021 Mar 18;16(3):e0248687. doi: 10.1371/journal.pone.0248687 (PMC7971507; doi:10.1371/journal.pone.0248687)
Supplement: S2 File — (DOCX) [file pone.0248687.s002.docx]

**Quality score based on the JBI assessment checklist for different study designs.**

1. **JBI Critical Appraisal Checklist for Studies Reporting Prevalence Data**
2. Was the sample frame appropriate to address the target population?
3. Were study participants sampled in an appropriate way?
4. Was the sample size adequate?
5. Were the study subjects and the setting described in detail?
6. Was the data analysis conducted with sufficient coverage of the identified sample?
7. Were valid methods used for the identification of the   condition?
8. Was the condition measured in a standard, reliable way for all participants?
9. Was there appropriate statistical analysis?
10. Was the response rate adequate, and if not, was the low response rate managed appropriately?

Based on these criteria, the quality score for prevalence study is given below.

| Author | Criteria and corresponding scores | | | | | | | | | Total | % |
| --- | --- | --- | --- | --- | --- | --- | --- | --- | --- | --- | --- |
|  | **#1** | **#2** | **#3** | **#4** | **#5** | **#6** | **#7** | **#8** | **#9** |  |  |
| Molla et al | 1 | 1 | 1 | 1 | 1 | 1 | 1 | 1 | 1 | 9 | 100 |

1. **JBI Critical Appraisal Checklist for Analytical cross-sectional studies**

| 1. Were the criteria for inclusion in the sample clearly defined? |
| --- |
| 1. Were the study subjects and the setting described in detail? |
| 1. Was the exposure measured in a valid and reliable way? |
| 1. Were objective, standard criteria used for measurement of the condition? |
| 1. Were confounding factors identified? |
| 1. Were strategies to deal with confounding factors stated? |
| 1. Were the outcomes measured in a valid and reliable way? |
| 1. Was appropriate statistical analysis used? |

Based on the above criteria, the quality score for analytical cross-sectional studies is given below.

| Author | Criteria and corresponding scores | | | | | | | | Total | % |
| --- | --- | --- | --- | --- | --- | --- | --- | --- | --- | --- |
|  | **#1** | **#2** | **#3** | **#4** | **#5** | **#6** | **#7** | **#8** |  |  |
| Mequanint et al | 1 | 1 | 0 | 1 | 0 | 1 | 1 | 1 | 6 | 75 |
| Wakjira et al | 1 | 1 | 1 | 1 | 0 | 1 | 1 | 1 | 7 | 87.5 |

1. **JBI Critical Appraisal Checklist for cohort studies**
2. Were the groups similar and recruited from the same population?
3. Were the exposures measured similarly to assign people to both exposed and unexposed groups?
4. Was the exposure measured in a valid and reliable way?
5. Were confounding factors identified?
6. Were strategies to deal with confounding factors stated?
7. Were the groups/participants free of the outcome at the start of the study (or at the moment of exposure)?
8. Were the outcomes measured in a valid and reliable way?
9. Was the follow up time reported and sufficient to belong enough for outcomes to occur?
10. Was follow-­up complete, and if not, were the reasons to loss to follow-up described and explored?
11. Were strategies to address incomplete follow-­up utilized?
12. Was appropriate statistical analysis used?

The quality measures of Cohort (retrospective and prospective) studies based on the above listed criteria

| Author | Criteria and corresponding scores | | | | | | | | | | | Total | % |
| --- | --- | --- | --- | --- | --- | --- | --- | --- | --- | --- | --- | --- | --- |
|  | **#1** | **#2** | **#3** | **#4** | **#5** | **#6** | **#7** | **#8** | **#9** | **#10** | **#11** |  |  |
| Alene et al | 1 | 1 | 1 | 0 | 1 | 0 | 1 | 1 | 1 | 0 | 1 | 8 | 72.73 |
| Baye et al | 1 | 1 | 1 | 0 | 0 | 0 | 1 | 1 | 1 | 0 | 0 | 6 | 54.55 |
| Fenataw et al | 1 | 1 | 1 | 0 | 1 | 0 | 1 | 0 | 0 | 0 | 1 | 6 | 54.55 |
| Girum et al | 1 | 1 | 1 | 0 | 1 | 0 | 1 | 1 | 1 | 1 | 0 | 8 | 72.73 |
| Kassa et al | 1 | 1 | 1 | 0 | 1 | 0 | 1 | 1 | 1 | 0 | 1 | 8 | 72.73 |
| Meressa et al | 1 | 1 | 1 | 0 | 1 | 0 | 1 | 1 | 1 | 0 | 1 | 8 | 72.73 |
| Shibre et al | 1 | 1 | 1 | 0 | 1 | 0 | 1 | 1 | 1 | 0 | 1 | 8 | 72.73 |
| Tola et al | 1 | 1 | 1 | 0 | 1 | 0 | 1 | 1 | 1 | 0 | 1 | 8 | 72.73 |
| Woldeyohans et al | 1 | 1 | 1 | 0 | 1 | 0 | 1 | 1 | 1 | 0 | 1 | 8 | 72.73 |

NB: 1 indicates the article does fulfill the specified criteria

0 indicates the article does not fulfill the stated criteria
