# Supplementary material for: Lost to follow-up and associated factors among patients with drug resistant tuberculosis in Ethiopia: A systematic review and meta-analysis
Source: PLoS One. 2021 Mar 18;16(3):e0248687. doi: 10.1371/journal.pone.0248687 (PMC7971507; doi:10.1371/journal.pone.0248687)
Supplement: S4 File — (DOCX) [file pone.0248687.s004.docx]

**WOLLO UNIVERSITY**

**COLLEGE OF MEDICINE AND HEALTH SCIENCES**

**SCHOOL OF PUBLIC HEALTH**


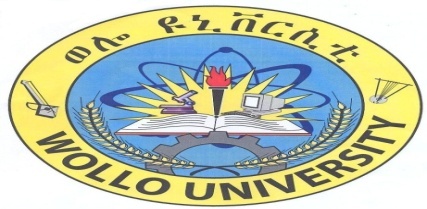


**Lost to follow-up and associated factors among patients with Multi-drug Resistant Tuberculosis in Ethiopia: A protocol to systematic review and metanalysis**

**Assefa Andargie**

**Asressie Molla**

**Fentaw Tadese**

**Segenet Zewdie**

**January 2020**

**Dessie, Ethiopia**

# **ACKNOWLEDGEMENTS**

I would like to thank Wollo University for the arrangement made from topic selection to this protocol finalization.

# **ABBREVIATIONS AND ACRONYMS**

AIDS Acquired immune deficiency syndrome

BCG Bacilli Calmette-Guerin

CI Confidence interval

DOT Directly observed treatment

HINARI Health Inter Network Access to Research Initiative

HIV Human immunodeficiency virus

JBI Joanna Briggs Institute

LTFU Lost to follow up

MDR-TB Multi-drug resistant tuberculosis

MeSH Medical Subject Headings

OR Odds ratio

PRISMA Preferred Reporting Items for Systematic Reviews and Meta-Analyses

PROSPERO Prospective register of systematic review and meta-analysis

PubMed Public/Publisher MEDLINE

RR Rifampicin resistance

RR-TB Rifampicin resistant Tuberculosis

TB Tuberculosis

US United States

WHO World health organization

XDR-TB Extensively drug resistant tuberculosis

**Table of Contents**

[**ACKNOWLEDGEMENTS** ii](#_Toc30147089)

[**ABBREVIATIONS AND ACRONYMS** iii](#_Toc30147090)

[**SUMMARY** v](#_Toc30147091)

[**1.** **INTRODUCTION** 1](#_Toc30147092)

[**1.1.** **Background** 1](#_Toc30147093)

[**1.2.** **Statement of the problem** 2](#_Toc30147094)

[**1.3.** **Significance of the review** 4](#_Toc30147095)

[**2.** **OBJECTIVES** 5](#_Toc30147096)

[**2.1.** **General objective** 5](#_Toc30147097)

[**2.2.** **Specific objectives** 5](#_Toc30147098)

[**3.** **METHODS** 6](#_Toc30147099)

[**3.1.** **Protocol registration and review reporting** 6](#_Toc30147100)

[**3.2.** **Eligibility criteria** 6](#_Toc30147101)

[**3.3.** **Information sources** 6](#_Toc30147102)

[**3.4.** **Search strategy** 7](#_Toc30147103)

[**3.5.** **Study records** 7](#_Toc30147104)

[**3.6.** **Data items** 7](#_Toc30147105)

[**3.7.** **Outcomes and prioritization** 8](#_Toc30147106)

[**3.8.** **Risk of bias in individual studies** 8](#_Toc30147107)

[**3.9.** **Data synthesis** 8](#_Toc30147108)

[**3.10.** **Meta-bias(es)** 9](#_Toc30147109)

[**3.11.** **Ethical considerations** 9](#_Toc30147110)

[**4.** **WORK PLAN** 10](#_Toc30147111)

[**5.** **BUDGET PROPOSAL** 11](#_Toc30147112)

[**REFERENCES** 12](#_Toc30147113)

[**Annex A: The PRISMA flow chart** 14](#_Toc30147114)

[**Annex B: Data extraction format** 15](#_Toc30147115)

# **SUMMARY**

**Introduction:** Drug-resistant Tuberculosis is caused by *Mycobacterium tuberculosis* organisms resistant to drugs typically used to treat the disease. Globally in 2018, 484,000 people developed tuberculosis that was resistant to rifampicin, and of these, 78% had multidrug-resistant tuberculosis. About 187,000 cases of multi-drug resistant tuberculosis were detected and notified in 2018. Among cases of multi-drug resistant tuberculosis in 2018, 6.2% were estimated to have extensively drug-resistant tuberculosis. In the year 2017, one third of global antimicrobial resistance deaths are attributed to multi-drug resistant tuberculosis. Treatment interruption is one of the causes of the development of acquired drug resistance tuberculosis. There are up to 22.3% treatment default rates in Sub Saharan Africa.

**Objective:** To review the pooled prevalence of treatment default and associated factors among multi-drug resistant tuberculosis patients in Ethiopia

**Methods:** a review of cross sectional, case control, cohort and analytical studies searched from PubMed, Scopus and HINARI will be performed from January to March 2020. Studies which report treatment default or lost to follow up and associated factors among adult population (age >18) will be included in the review. The Joanna Briggs Institute critical appraisal checklist will be used to evaluate the quality of primary researches and data will be extracted using Micro soft excel spreadsheet. Metanalysis will be performed using STATA Version 14. After examining heterogeneity of studies by forest plot and I^2^ heterogeneity test, fixed or random effect models will be employed. Sensitivity analysis will be performed to assess the contribution of individual studies and publication bias will be assessed using Eager’s test. The pooled estimates and effect measures will be reported using odds ratio and the 95% CI.

**Work plan and budget:** this review will be performed from January to April 2020 with a total of 7,485 ETB.

# **INTRODUCTION**

## **Background**

Tuberculosis (TB) is one of the most significant diseases in the history of humankind and remains an astonishing burden on human health today. Archaeological evidence reveals that tuberculosis was present in ancient times (1). Nonetheless, its cause continued indefinite until 1882, when Robert Koch discovered *Mycobacterium tuberculosis* bacillus. The bacillus estimated to be appeared around 70,000 years ago in Africa and then spread along with modern humans. The bacillus is a rod-shaped, non-spore-forming, and thin aerobic bacterium measuring 0.5μm by 3μm. It usually infects the lungs causing pulmonary TB but can also infect other sites of the body resulting in extrapulmonary TB. Tuberculosis is transmitted usually through the spread of droplet nuclei produced by patients with infectious pulmonary TB during sneezing, coughing, laughing and talking (2)**.**

TB is among the top 10 causes of mortality, and the principal cause of a single infectious agent. In 2018, there have been an estimated 10 million new TB cases worldwide. About 9% of the total account for people living with HIV. Eight countries: India, China, Indonesia, the Philippines, Pakistan, Nigeria, Bangladesh, and South Africa accounted for 66% of the new cases. An estimated 1.5 million people died due to TB. The distribution of the disease varies widely among countries. In most high-income countries, there were fewer than 10 new cases per 100,000 population. Whereas, in most of the 30 high TB burden countries, there were 150 to 400 incident cases per 100,000 population, and it was above 500 new cases per 100,000 in a few countries including Mozambique, the Philippines and South Africa (3).

Diagnostic tests for TB disease consist of sputum smear microscopy, rapid molecular tests, and culture-based methods. Effective drug treatments were first developed in the 1940s. The Bacilli Calmette-Guerin (BCG) is the only licensed vaccine for the prevention of TB disease. The vaccine was developed 100 years ago and prevents severe forms of TB in children and is widely used. At present, no vaccine is effective in averting TB disease in adults, either before or after exposure to TB infection (3).

## **Statement of the problem**

Drug-resistant TB is caused by the same organism as drug-susceptible TB but resistant to drugs classically used to treat the disease. It is transmitted similarly and is no more infectious than drug-susceptible TB. Considering the level of resistance, TB can be classified into various categories. Mono-resistance is resistance to one first-line anti-TB drug only. Poly-resistance is resistance to more than one first-line anti-TB drug, other than both isoniazid and rifampicin. Multidrug resistance (MDR): resistance to a minimum of both isoniazid and rifampicin. Extensive drug resistance (XDR) is resistance to any fluoroquinolone, and a minimum of one among the three second-line injectable drugs (capreomycin, kanamycin, and amikacin). Rifampicin resistance (RR) is resistance to rifampicin detected using phenotypic or genotypic methods, with or without resistance to other anti-TB drugs including any resistance to rifampicin, within mono-resistance, poly-resistance, MDR or XDR (4,5).

Globally in 2018, 484,000 people developed TB that was resistant to rifampicin (RR-TB), and of these, 78% had multidrug-resistant TB (MDR-TB). About 187,000 cases of MDR/RR-TB were detected and notified in 2018. Among cases of MDR-TB in the same year, 6.2% were estimated to have extensively drug-resistant TB (XDR-TB) (3). In the year 2017, one-third of global antimicrobial resistance deaths are attributed to MDR-TB (6). A study published in The Lancet Infectious Diseases forecasts that cases of drug-resistant TB will increase in four high burden countries (India, the Philippines, Russia, and South Africa) over the next 20 years (7).

According to a study based on reference laboratory data in Ethiopia, among 10,134 TB suspected individuals, 1,183 (11.7%) were culture positive. Among 329 Mycobacterium tuberculosis complex isolates by which first-line drug susceptibility test was performed, the proportion of resistance was 5.7% and 6.3% for isoniazid and rifampicin respectively. The proportion of multidrug-resistant tuberculosis (MDR-TB) was 4.3% in new patients, while 6.7% in previously treated patients and the overall proportion of MDR-TB was 11.6% (8).

TB that is resistant to first-line and second-line anti-TB drugs can be detected using rapid tests, culture methods, and sequencing technologies. Treatment for people with rifampicin-resistant TB (RR-TB) and multidrug-resistant TB (MDR-TB) is longer and requires drugs that are more expensive (≥US$ 1000 per person) and more toxic. Of the 187,000 cases of MDR-TB in 2018, a total of 156,000 were enrolled and started on treatment with a second-line regimen. The latest data reported to WHO show a treatment success rate for MDR-TB of 56% globally (3). About 54 million lives were saved between 2000 and 2017 through effective diagnosis and treatment (9).

Based on the treatment outcome, a patient may be categorized as cured, treatment completed, treatment failure, interrupter and loss to follow up. This study is interested to determine the magnitude of defaulters, currently known as lost to follow up. Treatment interrupters are patients who received treatment for at least 4 weeks and discontinued treatment for less than eight consecutive weeks. Defaulters or lost to follow up are patients who received treatment for at least 4 weeks and discontinued treatment for more than consecutive eight weeks (10).

According to a systematic review in Sub Saharan Africa, the treatment outcome among MDR-TB patients were 34.9% cure, 18.1% death, and a range of 1% to 22.3% treatment default (11). In South Africa, about 15.4% of rifampicin-resistant TB patients were lost to follow up (12). Similarly, in Peru, about 10% of patients have defaulted from treatment (13).

Many primary studies have identified factors associated with loss to follow up. A cross-sectional study in India identified age, sex, educational level and socioeconomic status to be associated with loss to follow up from TB treatment (14). Other factors like severity of resistance patterns, previous default and year of enrollment are also associated with treatment default (15). Substance use, substandard housing conditions, and health district are also predictors of treatment default among TB patients (13).

Finding from a systematic review and meta-analysis conducted in 2012 identified strategies associated with lower default rates. These strategies included the engagement of community health workers as directly observed treatment (DOT) providers, the provision of DOT throughout treatment, smaller cohort sizes and the provision of patient education (16).

The objectives of TB treatment are to cure the patient of TB, prevent death from TB disease and its late effects, prevent relapse of TB, prevent the development of acquired drug resistance, and decrease TB transmission in the community. Hence, Anti-TB treatment is said to be adequate when it is administered in the appropriate combination of drugs, in the correct dosage, regularly taken by the patient, and for a sufficient time (10).

Defaulting from treatment is one of the reasons for the development of acquired drug resistance. In MDR-TB treatment, interruption or loss to follow up will increase the probability of developing XDR-TB. There are many primary studies and systematic reviews and metanalyses conducted on the treatment outcome and associated factors of MDR-TB. The primary studies provide variations in the estimate of the prevalence and associated factors of treatment default among MDR-TB patients. The systematic reviews generally considered factors associated with good and poor treatment outcomes. Here the poor outcome included death, treatment failure, treatment default and/or loss to follow up. There is no pooled evidence on the factors associated, specifically, with treatment default which is an important outcome related to the development of acquired drug resistance and of course plays an important role in the transmission of MDR-TB within the community. Therefore, this review will attempt to bridge the gap in knowledge on the magnitude and the factors that are associated with treatment default among MDR-TB patients in Ethiopia.

## **Significance of the review**

Public health interventions will be effective when they are implemented to avoid root causes of health problems. Drug resistance tuberculosis is developed, on one hand, due to treatment non adherence such as defaulting or loss to follow up. Identification of the factors that predispose a person to interrupt MDR-TB treatment provides a basis for development of clinical and public health interventions. When the evidence is a pooled one, it becomes more valid and reliable for practice. Therefore, this review will provide a strong evidence for policy makers to reconsider the current TB treatment programs and guidelines. Clinicians will also get a better understanding on the factors that enhance or inhibit patients to default their treatment and hence to provide counselling on treatment adherence. The review also provides information on previous research gaps that should be studied further by researchers.

# **OBJECTIVES**

## **General objective**

To review the prevalence of treatment default and associated factors among patients with multi-drug resistant tuberculosis in Ethiopia

## **Specific objectives**

- To determine the pooled prevalence of MDR-TB treatment default in Ethiopia
- To estimate the pooled effect of factors associated with MDR-TB treatment in Ethiopia.

# **METHODS**

## **Protocol registration and review reporting**

Initially, databases were searched to check for the same systematic review in order to avoid duplicates. PubMed, Cochrane/Wiley Library and PROSPERO were explored to confirm whether previous systematic review and/or meta-analysis exists with the same topic. This systematic review and meta-analysis will be registered at the international prospective register of systematic review and meta-analysis (PROSPERO). The review will be conducted in accordance with the Preferred Reporting Items for Systematic Reviews and Meta-Analyses: The PRISMA Statement (17).

## **Eligibility criteria**

**Inclusion criteria**

- All observational studies including prevalence cross-sectional, analytical cross-sectional, case control and cohort studies conducted in Ethiopia.
- All articles published only in the English language regardless of any time specification.
- All studies that reported final outcomes and/or associated factors on treatment of adult culture-confirmed MDR-TB patients.

**Exclusion criteria**

- Studies that are conducted exclusively among children
- Studies which provide only interim outcomes (defined as ≤12 months on treatment) to avoid bias towards a lower default rate.
- Studies reporting outcomes on fewer than 50 patients and studies in which all patients had extensively drug-resistant tuberculosis.
- Studies with methodological limitations, such as incorrect outcome ascertainment criteria.

## **Information sources**

To access published primary studies, PubMed, Scopus and Health Inter Network Access to Research Initiative (HINARI) database sources will be used. To supplement the electronic data base searches, the online archives of the *International Journal of Tuberculosis and Lung Disease* will be reviewed for applicable studies. Grey literatures will be retrieved using Google and Google Scholar. In addition, the reference lists of the retrieved studies will be probed to collect articles that are not accessible through databases as well as electronic search engines. During the search process, to suppress the number of irrelevant studies, the search will be restricted to only ‘human studies’ and ‘English language’ in the advanced search. The corresponding author(s) will be contacted via mail or other means of communication for articles with full texts that are hard to access.

## **Search strategy**

The following are the key search terms that will be used in PubMed search.

1. “drug resistance” OR “multi drug resistance” OR MDR
2. Tuberculosis OR TB OR “MDR-TB”
3. “treatment outcome*” OR default* OR “treatment default” OR “loss to follow up”
4. “determinant*” OR factor* OR “associated factor*” OR predictor*
5. Ethiopia

In the advanced search of databases, the search strategy will be built based on the above-mentioned terms using the ‘Medical Subject Headings (MeSH)’ and ‘All fields’ by linking them with the “AND” Boolean operator i.e. #1 AND #2 AND #3 AND #4 AND #5

## **Study records**

The article search and screening activity will be done by AA and FT. Articles searched from different sources will be exported to EndNote V.7, and duplicates will be identified and removed. The remaining articles will be evaluated in the context of the topic, study participants, language and study area. Irrelevant topics, studies conducted out of Ethiopia and articles documented other than the English language will be rejected. The abstracts and full texts of the remaining studies will be reviewed. If the study’s full text cannot be accessed after an attempt to contact the original article author, they will be excluded.

## **Data items**

Once eligible studies are identified, two independent reviewers (AA and FT) will extract the relevant data using a pre-created format on Microsoft Excel spreadsheet. Information such as the primary investigator’s name, sample size, number of defaulted cases, response rate, study year, publication year, study setting/region, age groups of study participants (if applicable), study design and pertinent associated factors will be extracted. For prevalence studies prevalence, logarithm of prevalence and standard error (SE) of logarithm of prevalence will be computed. Similarly, for determinants odds ratio (OR), logarithms of OR and SE of the logarithms of OR will be computed. For any difficulties that might be encountered during data extraction, the corresponding author(s) will be contacted by any means of communication. When there are missing data, we will attempt to contact the original authors of the study to obtain the relevant missing data. Important numerical data will be carefully evaluated. If missing data cannot be obtained, an imputation method will be used.

## **Outcomes and prioritization**

The main outcome of this review is default or loss to follow up from MDR-TB treatment. According to WHO, treatment default is defined as treatment interruption for two or more consecutive months (8 weeks) for any reason without medical approval (18)**.** Currently, the term has been replaced by “Lost to follow up (LTFU)” which includes patient who has discontinued TB treatment for eight or more consecutive week after initiated on Anti-TB treatment (10).

## **Risk of bias in individual studies**

The quality assessment appraisal will be performed by two independent reviewers (AA and FT). The quality of each article will be assessed using the standardized Joanna Briggs Institute (JBI) critical appraisal tool prepared for cohort studies, case–control, cross-sectional and analytical cross-sectional studies. All tools have ‘Yes’ and ‘No’ types of questions, and scores will be given 1 and 0 for ‘Yes’ and ‘No’ responses, respectively. Scores will be summed and transformed into a percentage. Only studies that scored ≥50% will be considered for both systematic review and meta-analysis of prevalence of treatment default. When there are any scoring disagreements between the assessors, the sources of discrepancy will be investigated by a thorough discussion. For persistent disagreements in spite of the detailed review, a third independent reviewer (AM) will be assigned as arbitrator. Similarly, for determinants, each factor with each outcome variable will be critically appraised. The similar cut-off point that we will be using for prevalence studies will be applied to factors. Moreover, the quality results of primary studies will be placed in a separate column of the data extraction format.

## **Data synthesis**

The extracted data will be exported to STATA/SE V.14 for further analysis. The existence of heterogeneity among studies will be examined by I^2^ heterogeneity test. The I^2^ values of 25%, 50% and 75% will be interpreted as the presence of low, medium and high heterogeneity, respectively. Heterogeneity test (I^2^) of ≥50% and a p-value of <0.05 will be declared as the presence of heterogeneity. The Mantel-Haenszel method will be used for the fixed effect model if tests of heterogeneity are not significant. The presence of an association between the determinants and the outcome variables will be estimated based on the odds ratio (OR) with the respective 95% CI.

If statistical heterogeneity is observed (I^2^ >=50% or P <0.1), the DerSimonian and Laird random-effects model will be employed (19). Sensitivity analysis will be carried out to identify the influential studies that resulted in variation. Then, for extreme outlier studies, the extracted data will be checked for any error that might occur during the data extraction processes and if the data are free of errors, articles will be excluded from the analysis. Similarly, subgroup analyses will be employed by assuming the region, study design and the year of the study as grouping variables and sources of variation. If quantitative synthesis is not appropriate, a systematic narrative synthesis will be provided with information presented in the text and tables to summarize and explain the characteristics and findings of the included studies. The narrative synthesis will explore the relationship and findings both within and between the included studies.

## **Meta-bias(es)**

Publication bias will be detected by the funnel plot and Egger’s regression test (20). Accordingly, asymmetry of the funnel plot and/or statistical significance of Egger’s regression test (p<0.05) will be suggestive of publication bias. Therefore, a non-parametric trim and fill (Duval and Tweedie’s) method of analysis will be done (21). Using the Laird random-effects model, the pooled prevalence of treatment default will be reported.

## **Ethical considerations**

Ethical clearance letter will be obtained from the ethical review committee of college of medicine and health sciences, Wollo University after reviewed for the scientific appropriateness of the systematic review. Informed consent, privacy and confidentiality of the participants are assumed to be addressed by the primary researches and hence will not be applicable for this review.

# **WORK PLAN**

| **No** | **Activities** | **Time** | | | | | | | | | **Responsible body** |
| --- | --- | --- | --- | --- | --- | --- | --- | --- | --- | --- | --- |
|  |  | **Oct. 2019** | **Nov. 2019** | **Dec. 2019** | **Jan 2020** | **Feb. 2020** | **Mar 2020** | **Apr. 2020** | **May 2020** | **Jun.**  **2020** |  |
|  | Protocol development |  |  |  |  |  |  |  |  |  | PI |
|  | Protocol submission |  |  |  |  |  |  |  |  |  | PI |
|  | Protocol defense |  |  |  |  |  |  |  |  |  | PI |
|  | Ethical approval |  |  |  |  |  |  |  |  |  | CMHS |
|  | Study Search |  |  |  |  |  |  |  |  |  | PI, CI |
|  | Study selection |  |  |  |  |  |  |  |  |  | PI, CI |
|  | Data extraction |  |  |  |  |  |  |  |  |  | PI, CI |
|  | Data analysis |  |  |  |  |  |  |  |  |  | PI, CI |
|  | Write up of the findings (first draft) |  |  |  |  |  |  |  |  |  | PI |
|  | Write up of the findings (second draft) |  |  |  |  |  |  |  |  |  | PI |
|  | Write up of final report |  |  |  |  |  |  |  |  |  | PI |
|  | Mock defense |  |  |  |  |  |  |  |  |  | PI |
|  | Final defense |  |  |  |  |  |  |  |  |  | PI |
|  | Publication |  |  |  |  |  |  |  |  |  | PI, CI |

Where: PI: Principal Investigator

CMHS: College of medicine and health sciences (Ethical approval committee)

CI: Co-investigators

# **BUDGET PROPOSAL**

| **Personnel** | **Measurement** | **Unit cost (ETB)** | **Multiplying factor** | **Total** |
| --- | --- | --- | --- | --- |
| - Searcher and quality appraiser | Person | 200 | 2*2 | 800 |
| - Data extractor | Person | 200 | 2*3 | 600 |
| - Data analyzer | Person | 200 | 10 | 2,000 |
| **Sub total** | | | | 3,400 |
| **Supplies and services** |  |  |  |  |
| - Pen | Number | 10 | 3 | 30 |
| - Pencil | Number | 5 | 3 | 15 |
| - Paper | Pack | 200 | 4 | 800 |
| - Eraser | Number | 20 | 3 | 60 |
| - Printing | Page | 5 | 500 | 2,500 |
| **Sub total** | | | | 3,405 |
| Total | | | | 6,805 |
| Contingency | | | | 680 |
| **Grand total** | | | | **7,485** |

# **REFERENCES**

1. David A. Warrell, Timothy M. Cox, John D. Firth, Edward J., J R. MDB. Oxford Textbook of Medicine Volume 1. 4th ed. David A. Warrell, Timothy M. Cox, John D. Firth, Edward J., J R. MDB, editor. Oxford Press; 2003.

2. J. Larry Jameson, Dennis L. Kasper, Dan L. Longo, Anthony S. Fauci, Stephen L. Hauser JL. Harrison’s principle of internal medicine. 20th ed. New York: McGraw-Hill Education; 2018.

3. World Health Organization. Global Tuberculosis Report 2019. Geneva; 2019.

4. World Vision. Technical Guideline for Tuberculosis ( TB ) and TB-HIV Program Implementation. 2017;1–28.

5. The Economist Intelligence unit Limited. It’s Time to End Drug-Resistant Tuberculosis: The case for action. 2019.

6. The Global fund. Drug-resistant Tuberculosis [Internet]. 2019. Available from: http://www.theglobalfund.org

7. Knight GM, Mcquaid CF, Dodd PJ, Houben RMGJ. Global burden of latent multidrug-resistant tuberculosis : trends and estimates based on mathematical modelling. Lancet Infect Dis [Internet]. 2019;19(8):903–12. Available from: http://dx.doi.org/10.1016/S1473-3099(19)30307-X

8. Diriba G, Kebede A, Tola HH, Alemu A, Tadesse M, Tesfaye E. Surveillance of drug resistance tuberculosis based on reference laboratory data in Ethiopia. Infect Dis Poverty. 2019;8(54):4–9.

9. World Health Organization. Global strategy and targets for tuberculosis prevention , care and control after 2015. 2018.

10. FDRE Ministry of health. NATIONAL COMPREHENSIVE TUBERCULOSIS , LEPROSY AND TB / HIV TRAINING MANUAL for HEALTH CARE WORKERS: PARTICIPANTS ‘ MANUAL. Addis Ababa; 2016. p. 45.

11. Chem ED, Hout MC Van, Hope V. Treatment outcomes and antiretroviral uptake in multidrug-resistant tuberculosis and HIV co-infected patients in Sub Saharan Africa : a systematic review and meta-analysis. BMC Infect Dis. 2019;19(723):1–8.

12. Id KH, Berhanu R, Evans D, Rosen S, Sanne I, Long L. High rates of death and loss to follow-up by 12 months of rifampicin resistant TB treatment in South Africa. PLoS One. 2018;13(10):1–13.

13. Shelke, A. R. , Roscoe, J. A. , Morrow, G. R. , Colman, L. K. , Banerjee, T. K. , & Kirshner JJ. Risk Factors and Mortality Associated with Default from Multidrug-Resistant Tuberculosis Treatment. Bone [Internet]. 2008;23(1):1–7. Available from: https://www.ncbi.nlm.nih.gov/pmc/articles/PMC2688697/pdf/nihms-110443.pdf

14. Pasha MAM, Fatima A, Gopichand S, Sushma M. A socio-demographic study of the “ loss to follow – up in TB cases under DOTS ” in and around tertiary teaching care hospital. Int J Community Med Public Heal. 2017;4(9):3123–8.

15. Lalor MK, Greig J, Allamuratova S, Althomsons S, Tigay Z, Khaemraev A, et al. Risk factors associated with default from multi- and extensively drug-resistant tuberculosis treatment, Uzbekistan: A retrospective cohort analysis. PLoS One. 2013;8(11).

16. Toczek A, Cox H, Cros P, Cooke G, Ford N. Strategies for reducing treatment default in drug-resistant tuberculosis : systematic review and meta-analysis. Int J Tuberc Lung Dis. 2012;17(August):299–307.

17. Moher D, Liberati A, Tetzlaff J, Altman DG, Group TP. Preferred Reporting Items for Systematic Reviews and Meta-Analyses : The PRISMA Statement. PLoS Med. 2009;6(7):1–6.

18. WHO. Definitions and reporting framework for tuberculosis – 2013 revision [Internet]. World Health Organization. 2014. 1–47 p. Available from: http://apps.who.int/iris/bitstream/10665/79199/1/9789241505345_eng.pdf

19. DerSimonian R, Laird N. Meta-analysis in clinical trials. Control Clin Trials. 1986;7(3):177–88.

20. Higgins JPT, Thompson SG. Quantifying heterogeneity in a meta-analysis. Stat Med. 2002;21(11):1539–58.

21. Duval S, Tweedie R. A Nonparametric “Trim and Fill” Method of Accounting for Publication Bias in Meta-Analysis. J Am Stat Assoc. 2000;95(449):89–98.

# **Annex A: The PRISMA flow chart**

Records identified through database searching
(n = )

Additional records identified through other sources
(n = )

Records after duplicates removed
(n = )

Records screened
(n = )

Records excluded
(n = )

Full-text articles assessed for eligibility
(n = )

Full-text articles excluded, with reasons
(n = )

Studies included in qualitative synthesis
(n = )

Studies included in quantitative synthesis (meta-analysis)
(n = )

Identification

Included

Eligibility

Screening

# **Annex B: Data extraction format**

| **Author** | **Year of study** | **Study area** | **Study design** | **Sample size** | **Default** | **Other outcomes** | **Factors associated with default** | **Quality score** |
| --- | --- | --- | --- | --- | --- | --- | --- | --- |
|  |  |  |  |  |  |  |  |  |
|  |  |  |  |  |  |  |  |  |
|  |  |  |  |  |  |  |  |  |
|  |  |  |  |  |  |  |  |  |
|  |  |  |  |  |  |  |  |  |
|  |  |  |  |  |  |  |  |  |
|  |  |  |  |  |  |  |  |  |
|  |  |  |  |  |  |  |  |  |
|  |  |  |  |  |  |  |  |  |
