# Supplementary material for: Lost to follow-up and associated factors among patients with drug resistant tuberculosis in Ethiopia: A systematic review and meta-analysis
Source: PLoS One. 2021 Mar 18;16(3):e0248687. doi: 10.1371/journal.pone.0248687 (PMC7971507; doi:10.1371/journal.pone.0248687)
Supplement: Attachment — Submitted filename: Response to Reviewers.docx [file pone.0248687.s007.docx]

Assefa Andargie

Wollo University

Dessie, Ethiopia

[assefaand@gmail.com](mailto:assefaand@gmail.com)

PLOS one

February 10, 2021

**Response to points raised by the academic editor**

1. We have checked each section and all are inline with the PLOS ONE’s style requirement.
2. We have discussed how the current manuscript advances on the stated previous work in the introduction section of the manuscript (page 5, line 92-100)
3. We included an additional text on the previous document to correlate each number to the listed assessment criteria (S2_file)
4. The publication date range used to find articles included in the methods section (page 7, line 129).
5. Financial disclosure stated as ‘The authors received no specific funding for this work’ (page 21, line 394)
6. The cited table i.e., ‘Table 3’ in the text was replaced by ‘Table 2’ (page 15, line 284).
7. The cited table was wrong (editorial problem) and corrected as stated in number 6 above.

**Response to reviewers’ comments**

Reviewer #1

- The 8% LTFU was stated as high by comparing it with the aspired global target of 90% treatment success. It is corrected as considerable (page 18, line 332).

1. The study design labeled as ‘cohort’ was directly taken from the articles. As the reviewer raised, there is no any true cohort study that compared exposed and unexposed groups with respect to the outcome. But there are cohort analyzed studies focusing only a single group to determine the outcome. Therefore, what we could do as systematic review is to write the design that the primary researcher wrote.
2. The references list was corrected to fit with the journal requirement (page 24-25, line 475-489).

Reviewer #2

1. The cited table was changed from Table 3 to Table 2 (page 15, line 284)
2. Figure 5 was available. We do not know whether the copy sent to this reviewer had a problem. Still, it is attached as a separate file labeled as ‘Fig5’.
3. Regarding few numbers of articles cited in the discussion while there were 13 references in the introduction,

Most of the citations in the introduction were global reports and documents other than research articles. There was a shortage of similar reviews on LTFU which could be used in the discussion section. That is why we compared our finding the global target. Therefore, it is due to the lack of similar findings in line with our review that forced us to cite only 2 references in the discussion section. We have added 2 more references in the discussion to support our explanation about the finding (page 18, line 340 and page 19, line 351)

Reviewer #3

We have added references that support our explanation (page 18, line 340 and page 19, line 351)

In the limitation section, we added the importance of knowing the moment of LTFU and our inability to include this information in our analysis (page 20, line 373-376).

Thank you for your concern!

Sincerely,

Assefa Andargie

Lecturer, School of public health

Wollo University
